# Role of psychomotricity in the management of body image disorders in schizophrenia: a case report

**DOI:** 10.11604/pamj.2021.40.184.27107

**Published:** 2021-11-26

**Authors:** Christian Eyoum, Nathalie Kingue Mbenda, Rodrigue Tchokona Kontchou, Simon Noé Elessa Belle, Erero Njiengwe

**Affiliations:** 1 Department of Psychiatry, Laquintinie Hospital, Douala, Cameroon,; 2 African Leadership College, Pamplemousses, Mauritius,; 3 Garoua Regional Hospital, Garoua, Cameroon,; 4 Institut Convergence Psy-Santé, Douala, Cameroon

**Keywords:** Psychomotricity, schizophrenia, body image, fragmentation, case report

## Abstract

Schizophrenia is one of the most debilitating psychiatric disorders affecting around 1% of people worldwide. Its causes and management are quite poorly controlled. Patients with schizophrenia often experience an alteration in their body image. Its corollaries such as depersonalization are felt like real torture. In the biopsychosocial model of the management of mental health disorders, very few tools are effective in the management of depersonalization syndrome which is often overlooked by psychiatrists who mainly focus on erasing hallucinations and other positive symptoms. Psychomotricity, a poorly known branch of the biopsychosocial model, is still trying to find a place between psychological and body therapies. For a period of 6 months, we conducted a prospective case-study on two patients living with schizophrenia and treated in the Psychiatry Department of Laquintinie Hospital in Douala in Cameroon. In those patients, the association of psychomotor therapies provided a satisfactory response to a problem of depersonalization, also known as fragmentation anxiety.

## Introduction

Schizophrenia is a chronic psychosis comprised by the following triad: positive symptoms, negative symptoms and cognitive symptoms [1]. Schizophrenia affects 1% of people in the world regardless of their race, and approximately 250,000 people in Cameroon. The disorder's first symptoms usually appear between the ages of 15 and 35.

Its management is based on the recommendations of the World Health Organization and on the biopsychosocial model. We observe, globally, in many cases, a relapse due to partial adherence to the treatment, to poorly tolerated side effects of the treatment and to the lack of social support to patients. The discovery of neuroleptics (first generation antipsychotics) in France in the middle of the 20^th^ century gave mental health an essential tool in the management of this constellation, but also contributed to put to oblivion therapeutic models which, in the shadow of drugs, have long brought under control the clinical signs of schizophrenia [2].

Following the body-mind approaches stated in the psycho-corporal axis [3], this article reports a prospective case-study carried out between October 30^th^, 2016 and April 30^th^, 2017, in the Psychiatry Department of Laquintinie Hospital in Douala, Cameroon. The two patients we selected, were diagnosed with schizophrenia according to the criteria of the 10^th^ edition of the International Classification of Diseases. In fact, these two patients presented a severe anxiety of fragmentation, which was the main criteria of their selection in our study.

## Patient and observation

### Patient 1

**Patient information:** Mr. X, 26-year-old, is a writer who currently lives with his parents. He is the eight among 13 full siblings. He is single with no children. Mr. X arrived at the interview with a sad, poor and inexpressive mimic. He came for a consultation accompanied by his mother. The main complaints were insomnia and psychomotor agitation at home. The diagnosis proposed was that of a relapse of schizophrenia. The patient has a familial history of schizophrenia. Actually, his mother and brother are followed-up in the same psychiatric department. Mr X. has no history of substance use. The recent family dynamic is tinged with some altercations, in particular between the patient and his father. At the time we proposed to Mr. X to go through a psychomotor evaluation, the positive and negative symptoms seemed relatively under control.

**Figure 1:**
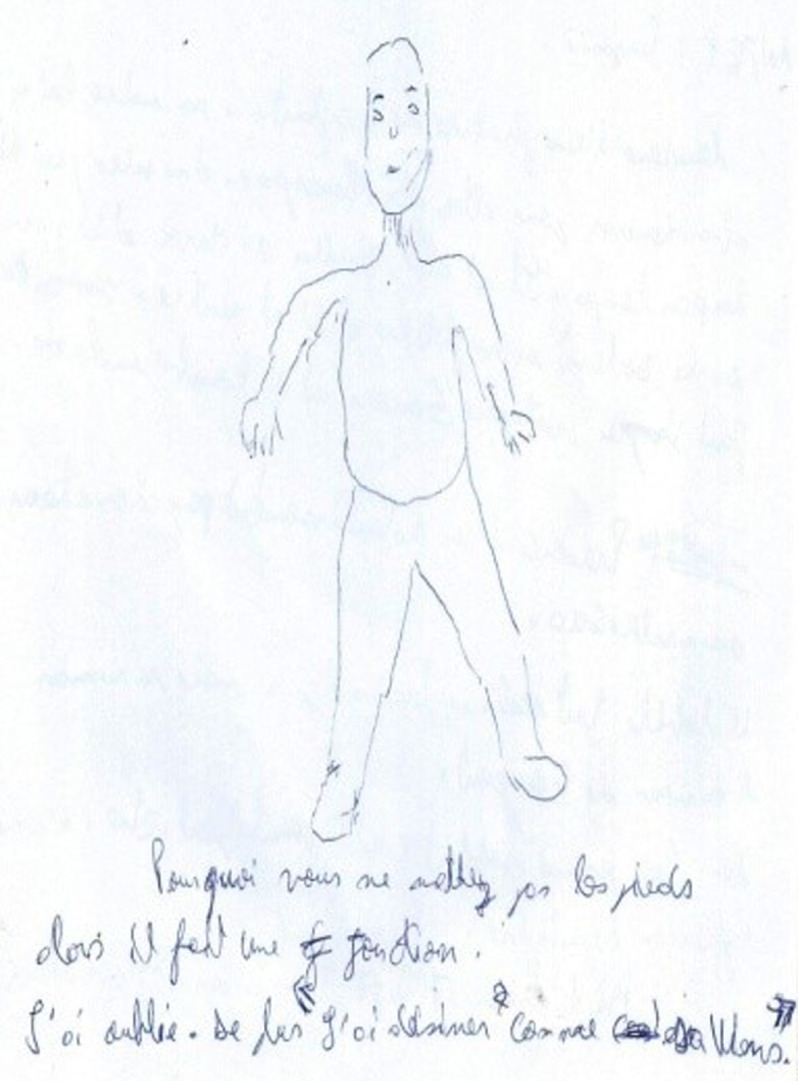
patient 1 (Mr. X) drew himself before treatment, no joints

**Figure 2:**
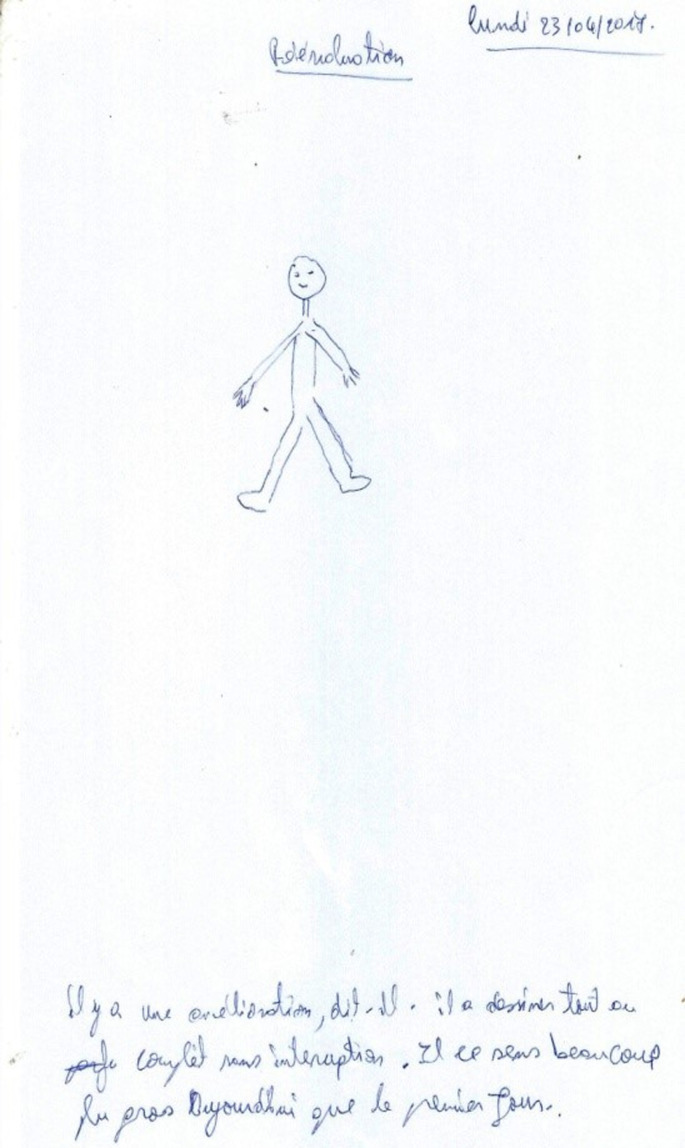
patient 1 (Mr. X) drawing after one month treatment

**Clinical findings:** concerning muscle tone, we observe the presence of a Dupré-type paratonia at the outstretched arms test and the absence of puppets overlays. Movements are slow and not easy to perform. Regarding the fine motor skills, on Rey's digital test, all fingers move upward on the right hand at the same time while on the left they don't. The thumb is easier to move on the right hand than on the left hand. Regarding the body diagram and body image, the reproduction of head´s figures is done in mirror. On the man's drawing, he draws the head, then looks at his hands and says: “I cannot draw myself!”. After his mother´s insistence, he continues with the drawing of the hands, then stops and tells us that his body is made out of lead and he does not perceive all the parts of his body (Figure 1, Figure 2). Concerning spatial tests, on the trajectory on the ground test, he fails to perform the first figures; at Soubiran's house he has difficulty with orientation. At Mira Stambak's rhythms test, he sometimes multiplies his taps and no longer knows the time frame (tomorrow and yesterday). In the market game, Mr. X names five objects regardless of the order.

**Timeline of the current episode:** at the entrance, *patient 1* presented the Dupré type paratonia; as well as hand-eye incoordination. On day 2, we started treatment. For *patient 1*, we opted for relaxation through a body and verbal mediated technique; Then on day 15, *patient 1* had a decrease in paratonia. On day 45, we noticed an improvement in hand-eye coordination for *patient 1*.

**Diagnostic assessment:** upon arrival in their respective wards, our patients underwent the basic assessment check-ups for the patients admitted to our unit. For the radiological examination, only *patient 1* was able to benefit from a brain scan which came back with abnormal findings. Patient 1 patients underwent a prolonged electroencephalogram which came back with no specific findings. In terms of biological assessments, the protocol of our service provides the following basic assessments: blood count, fasting blood sugar, transaminases (SGOT, SGPT), uremia, serum creatinine, thick smear for malaria check. These assessments came back with no specific findings.

**Therapeutic intervention:** we followed the principles of the bio-psycho-social model. On the biological level, patient 1 received antipsychotics for the management of schizophrenia. In terms of psychotherapy, *patient 1* benefited from supportive psychotherapy which was provided by the psychiatrist. On the psychomotor level, we used mediation techniques such as: relaxation (a body and verbal mediated technique acting on an individual through the body or the psyche in order to promote muscle and psychic relaxation resulting to body well-being); therapeutic touch (a body-mediated therapy acting on the body and promoting psycho-corporal well-being); and body awakening also known as body expression (therapy based on the use of the body in conjunction with space and time parameters, with the aim to bring out its value: dance, drama, role play…).

A psychomotor treatment session always begins with building the therapeutic bond between the patient, his close entourage and the therapist. As Gazon [4] explains, “insurance policies” must be put in place to gain access to someone's body, this is even more true in the context of a psychotic disorder where anxiety and distress are present more than ever [5]. When the patient arrives in our services we welcome and install him. After this first contact, we prepare him for about 10 minutes prior to the induction phase; we assess his mood and state of being. When it is done, we install the patient, for 20 minutes, on a dorsal decubitus position to induce relaxation, which can be done either by touch or verbal induction according to the patient´s preference. Then we proceed with body awakening through dancing, singing, role playing for a duration of 10 minutes. At last, we end with 5 minutes debrief of our session to get to know his feelings and thoughts during the therapy. The therapy session lasts 45 minutes on average (30 minutes at least and 60 minutes maximum).

**Follow-up and outcomes:** as demonstrated in the timeline above, once psychomotor management was put in place, we noted a marked improvement in muscle tone for both patients at the end of the second week, with a decrease in hypertonicity of the flexors in particular. At week 5, *patient 1* general dynamic coordination improved significantly.

### Patient 2

**Patient information:** Ms. Y is a 27-year-old hairdresser living in the city of Douala. She is a single mother of one child. She is the second born of three siblings. She came to consultation with her older brother. Her medical history is not relevant. The current symptomatology is centered on positive symptoms made of delusions, backed by auditory hallucinations, with the patient's strong adhesion. The diagnosis of paranoid schizophrenia was found.

**Figure 3:**
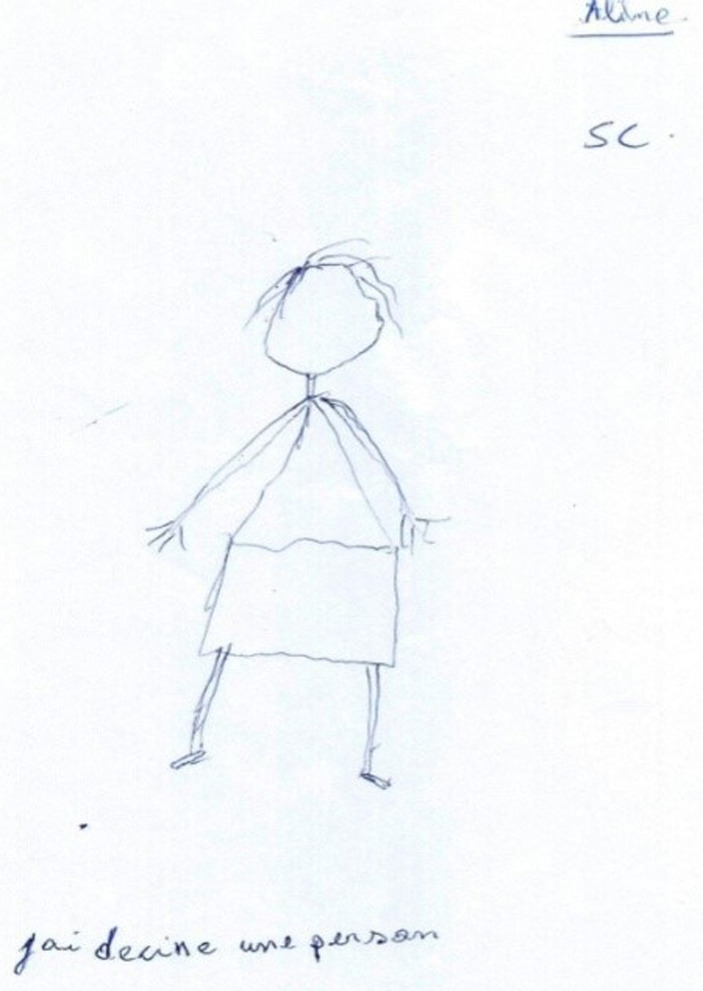
patient 2 (Ms. Y) drawing herself before treatment

**Figure 4:**
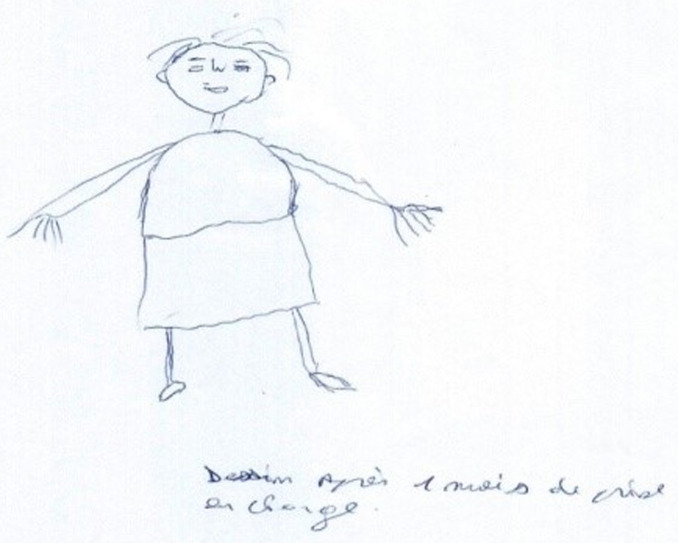
patient 2 (Ms. Y) drawing herself after one month treatment

**Clinical findings:** concerning muscle tone, there is resistance to performing movements. Regarding fine motor skills, Ms. Y can easily untie her fingers, but a tremor is observed when performing the thumb-finger test. Regarding the evaluation of the body diagram and body image, on the draw-a-Person test, there are several missing details such as eyes, nose, mouth, breasts, etc. (Figure 3, Figure 4). On the somatognosia test, she recognizes most of the body parts mentioned. On the Soubiran's muscle sense, the execution of the figures is done in mirror. Concerning the spatio-temporal evaluations, on the execution of the figure of Rey, the grip is tridigital. As she draws, she turns the sheet in all directions and begins to draw on it from the outside to the inside. Ms. Y does not know the great moments of her church, but she is aware of the date and the seasons. Ms. Y's visual-spatial memory is very confused.

**Timeline of the current episode:** *patient 2* had resistance to performing the movements and a tremor on the thumb-finger test. For her, we used therapeutic touch also called body awakening. On day 15, *patient 2* had a decrease in flexor hypertonia. On day 45 *patient 2*, we saw a regression of the spatio-temporal disorder (test of the house of Soubiran, figure of Rey).

**Diagnostic assessment:** upon arrival in their respective wards, our patients underwent the basic assessment check-ups for the patients admitted to our unit. Ms. Y could not get a brain computed tomography scan due to lack of funds. *Patient 2* patients underwent a prolonged electroencephalogram which came back with no specific findings. For the following basic assessments (blood count, fasting blood sugar, transaminases, uremia, serum creatinine, thick smear for malaria check), *patient 2* presented a mild, normochromic, normocytic anemia at 10g/dl which was corrected with an oral treatment.

**Therapeutic intervention:** on the biological level, *patient 2* received antipsychotics for the management of psychosis and her mild anemia was also corrected. In terms of psychotherapy, at that time, no patient could not receive any specified psychotherapy due to the lack of clinical psychologists within the department. *Patient 2* then benefited from supportive psychotherapy. On the psychomotor level, for *patient 2*, we used the same mediation techniques as for *patient 1*: relaxation; therapeutic touch; and body awakening also known as body expression. These psychomotor therapies are practiced in a multidisciplinary way with other therapies such as: psychotherapies, musicotherapy, hydrotherapy etc.

**Follow-up and outcomes:** at week 6, *patient 2* general dynamic coordination showed a markedly improved score. The figures show indirect evidence of improvement in our two patients (Figure 1, Figure 2, Figure 3, Figure 4). The follow-up lasted 6 months in order to ensure consolidation of the treatment and thus limit the risk of relapse in the long term.

## Discussion

Even since the discovery of neuroleptics in 1952, the management of mental illnesses and schizophrenia in particular has been consistently done through drug consumption. Up to now, psychotherapy is barely used. The bodily manifestations of the disease, especially body anxiety, is often overlooked. Sometimes and mostly in sub-Saharan Africa, they are attributed to witchcraft or mystic forces. The presence of these disorders undoubtedly places psycho-corporal therapies at the heart of the debate in the management of schizophrenia. Indeed, some psychomotor disorders (muscle tone, inhibition, coordination…) are caused by the drugs´ side effects, in that case, their remission, most often depends on reducing drugs dosage or on their cessation. However, other psychomotor disorders (memory, body image, mind-body unity, etc.) started during the different stages of an individual's psychomotor development.

It is theorized that in the pre-morbid states of psychosis, a latent anxiety of fragmentation slumbers within the subject's symbolic body [6]. This anxiety symbolically segments his body and is at the core of the disorders of the body-mind unit. Therefore, the body would only wait for a traumatic situation to trigger the disease. Psychic (Schulz's autogenic training), body (dance) and psycho-body (Soubiran's relaxation) mediations have an impact on the person with the aim of restoring him to a state of well-being [7]. In verbal-induced relaxation like Schulz's autogenic training (where the signal is first auditory before becoming somatic) or in Soubiran's psycho-corporal relaxation, it is the repetition of sessions that fosters the improvement of the patient's health.

The impact of these mediations on body image and therefore on self-esteem is undeniable. The importance of this is no longer to be demonstrated, when we know that in people with schizophrenia, the support strategy's ultimate goal is autonomy, which requires social empowerment. In this study, the therapeutic touch (TT) has proven to be very useful, helping to improve the well-being of our patients from the first sessions. This is in line with the work carried out by Ünal Aslan *et al*. [4] who showed that TT has a positive impact on spiritual wellness and on sleep quality of patients. After conducting a review of the literature on TT, covering the period from January 2009 to March 2020, Garett and Riou [5] identified 21 studies. Eighteen of those studies showed a positive impact of TT on psychiatric illnesses. In the work conducted by Vural Doğru *et al*. [6], out of 96 students divided into three groups, the group which went through TT came out with better results: reduced stress and improved quality of sleep.

Relaxation is another weapon against anxiety. Yuniartika in a case-control study in the elderly showed that relaxation is effective for sleep in the elderly [7]. However, Melo-Dias *et al*. out of 1172 selected studies retained 05 studies which showed (on 216 patients) a significant improvement on anxiety, well-being and social functioning. These results were achieved through progressive muscle relaxation (PMR) [8]. Lastly, in Taiwan [9], 80 patients in a psychiatric ward were separated into two groups. The group which in addition to the classic treatment received the progressive muscle relaxation (PMR) showed early and profound control of anxiety and psychotic symptoms.

## Conclusion

Psychomotricity remains to this day a discipline that is poorly understood and underused in mental health treatment protocols. However, the body is, as we know, the receptacle of the mind and the first connection between man and his external environment. The management of the body in order to rebuild the body-mind unity should therefore be a priority tool for supporting people with schizophrenia. The results obtained from the two cases of our study sufficiently illustrate the potential benefits from psychomotor therapies.

**Informed consent:** as a prerequisite to the study, we obtained the informed consent of each patient and their support person who agreed to participate, with respect of confidentiality.
